# ILDIM-MFAM: interstitial lung disease identification model with multi-modal fusion attention mechanism

**DOI:** 10.3389/fmed.2024.1446936

**Published:** 2024-11-18

**Authors:** Bin Zhong, Runan Zhang, Shuixiang Luo, Jie Zheng

**Affiliations:** ^1^Department of Respiratory Medicine, The First Affiliated Hospital of Gannan Medical University, Ganzhou, Jiangxi, China; ^2^College of Pharmacy, Gannan Medical University, Ganzhou, Jiangxi, China; ^3^Department of Nephrology, The First Affiliated Hospital of Gannan Medical University, Ganzhou, Jiangxi, China

**Keywords:** multimodal medical information, interstitial lung disease (ILD), multimodal fusion attention mechanism (MFAM), multimodal data sources, comprehensive assessment

## Abstract

This study aims to address the potential and challenges of multimodal medical information in the diagnosis of interstitial lung disease (ILD) by developing an ILD identification model (ILDIM) based on the multimodal fusion attention mechanism (MFAM) to improve the accuracy and reliability of ILD. Large-scale multimodal medical information data, including chest CT image slices, physiological indicator time series data, and patient history text information were collected. These data are professionally cleaned and normalized to ensure data quality and consistency. Convolutional Neural Network (CNN) is used to extract CT image features, Bidirectional Long Short-Term Memory Network (Bi-LSTM) model is used to learn temporal physiological metrics data under long-term dependency, and Self-Attention Mechanism is used to encode textual semantic information in patient’s self-reporting and medical prescriptions. In addition, the multimodal perception mechanism uses a Transformer-based model to improve the diagnostic performance of ILD by learning the importance weights of each modality’s data to optimally fuse the different modalities. Finally, the ablation test and comparison results show that the model performs well in terms of comprehensive performance. By combining multimodal data sources, the model not only improved the Precision, Recall and F1 score, but also significantly increased the AUC value. This suggests that the combined use of different modal information can provide a more comprehensive assessment of a patient’s health status, thereby improving the diagnostic comprehensiveness and accuracy of ILD. This study also considered the computational complexity of the model, and the results show that ILDIM-MFAM has a relatively low number of model parameters and computational complexity, which is very favorable for practical deployment and operational efficiency.

## 1 Introduction

Lung diseases have been one of the major contributors to serious health problems in modern medicine (1). Among them, Interstitial Lung Disease (ILD) is a group of diseases involving the interstitial of the lungs, including various types such as pulmonary fibrosis and alveolar protein deposition, etc. They are characterized by damage to the interstitial structures of the lungs, usually leading to symptoms such as dyspnea, cough, chest pain, and in severe cases, can even be life-threatening (2). The early diagnosis and effective management of ILD are crucial for improving the survival rate and quality of life of patients (3).

The diagnosis of ILD has always been one of the difficult problems in the medical community because of its complex etiology, diverse symptoms, and wide variability in presentation between patients (4). Traditional diagnostic methods for ILD rely on clinical symptoms, pulmonary function tests, and unimodal medical imaging data, such as chest CT images (5). However, these methods have some limitations, for example, they struggle to provide enough information to accurately diagnose different types of ILD, leading to an increased rate of misdiagnosis (6). Therefore, the development of an accurate and reliable method for recognizing ILD is essential for improving patient treatment and management.

With the rapid development of deep learning and computer vision technologies and their wide application, learning inference based on image modality has been widely used in both industrial and medical fields (7–10). Currently, a large number of studies have focused on the diagnosis of ILD, mainly on the application of unimodal medical information data, for example, chest CT images have become one of the key tools for ILD diagnosis. Its high resolution and detailed presentation of lung structures have made it the first choice for physicians (11). However, a single CT image modality still has some shortcomings. First, CT images cannot provide enough information to differentiate between different types of ILDs in some cases, as it relies heavily on the visibility of morphological features (12). Moreover, the higher radiation dose can pose a potential risk to the patient’s health, especially if multiple repeat examinations are required (13). In addition, unimodal physiologic index data, such as pulmonary function tests and blood biochemical indices, although they have applications in the adjunctive diagnosis of ILD, they are similarly limited because they do not provide direct information about the structure of the lungs (14). Therefore, existing unimodal ILD diagnostic methods, although helpful to some extent in physician decision-making, still have challenges and limitations in recognizing different ILD types. This has led to the emergence of multimodal medical information data in ILD diagnostic research, with the aim of being able to synthesize information from different modalities to improve the accuracy and reliability of ILD.

With the continuous progress in the fields of medical imaging, bioinformatics and artificial intelligence, the application of multimodal medical information data in the diagnosis and treatment of diseases has become a hot research topic of great interest (15–18). Multimodal medical information data include multiple types of medical data, such as images (19), time-series data (20), and textual information (21), which can provide comprehensive information about a patient’s health status. In the field of lung diseases, the application of multimodal data has already achieved some impressive results in the detection of lung cancer and lung nodules. Moreover, for the diagnosis of ILD, multimodal medical information data has always had great potential (22). First, multimodal data can provide physicians with a more comprehensive view to capture the characteristics of ILD from different dimensions, including structural, functional, and biochemical information (23). By combining chest CT images, physiologic index time series data, and textual information of patient history can more accurately portray the pathological changes and trends of ILD (24). Moreover, the comprehensive analysis of multimodal data can help distinguish different types of ILD, because different types of ILD can exhibit unique features on different modal data (25). This can help improve the precise classification and personalized treatment of ILD. In addition, multimodal data can be used to track the progress of ILD and monitor the treatment effect, providing better long-term management for patients (26). However, there are a number of challenges to realizing the potential of multimodal ILD diagnosis. First, fusion and correlation analysis between different modality data requires complex algorithms and models to handle to ensure synergy between various information (27). Moreover, data quality and accuracy are critical for multimodal ILD diagnosis and require effective data cleaning and preprocessing methods.

Given the potential and challenges of multimodal medical information data in ILD diagnosis, this study aims to develop an advanced ILD recognition model based on the multimodal fusion attention mechanism (MFAM) to improve the accuracy and reliability of ILD. By comprehensively utilizing chest CT image slices, physiological indicator time-series data, and textual semantic data, the model explored new possibilities in the field of ILD diagnosis and provide physicians with more comprehensive and accurate ILD diagnostic support to facilitate early diagnosis and effective treatment of lung diseases. First, we collect large-scale multimodal medical information data, including chest CT image slices, physiological indicator time-series data and patient history text information. These datasets come from different medical institutions and databases, and can be carefully cleaned and standardized to ensure the quality and consistency of the data. In addition, we also protected the privacy of the data to ensure the security of the patient’s sensitive information. MFAM is used to synthesize and learn different modal data to capture the multifaceted features of ILD. In which, CNN is used to extract CT image features, bidirectional LSTM (Bi-LSTM) model is used to learn temporal physiological index data under long-term dependency, and self-attention mechanism is used to encode textual semantic information with patient’s self-report and medical advice. Moreover, the multimodal perception mechanism employs a Transformer-based model to improve the diagnostic performance of ILD by learning the importance weights of each modal data so that the different modal data can be fused in an optimal way. Therefore, in the field of ILD diagnosis, this study has the following main technical contributions:

(1) Firstly, the problem of multimodal medical data fusion is solved by organically combining textual information, CT images, and physiological indicators information to describe the patient’s health status in a more comprehensive and multifaceted way.
(2) Furthermore, by adopting an attention mechanism to adaptively fuse information from different modalities, the model makes full use of the advantages of various data sources and fully considers the multidimensional features of ILD diagnosis, including structure, function and clinical history.
(3) Ultimately, the experimental results show that the model performs well in terms of comprehensive performance. By combining multimodal data sources, the model not only improves the precision, recall and F1 score, but also significantly increases the AUC value. This indicates that the combined use of different modal information can assess the health status of patients more comprehensively, thus improving the diagnostic comprehensiveness and accuracy of ILD.

## 2 Methodology

ILD patients can exhibit marked differences in physiologic and CT image characteristics compared with normal lungs. Physiologically, patients with ILD have decreased lung function, dyspnea, and abnormal digital indicators. On CT images, features such as ground-glass infiltrates, parenchymal infiltrates, bilateral distribution, and cobwebby or honeycomb fibrosis appear. These features are crucial for the diagnosis of ILD and the evaluation of the disease. Although clinical data reflected purely on physiologic or CT images can provide physicians with a good basis for diagnosis, relying solely on physiologic data or CT images can limit the comprehensiveness and accuracy of the diagnosis because they do not provide enough information to fully assess the condition of patients with ILD. Therefore, it is necessary and important to use both clinical data and CT images as inputs for a more accurate diagnosis and assessment of ILD.

**A. Introduction to the framework of the proposed model**

The purpose of this study was to develop a multimodal medical information diagnostic model to improve the accuracy and reliability of interstitial lung disease (ILD). The model is based on the Multimodal Fusion Attention Mechanism (MFAM) and involves multiple sources of medical information, including CT images, time-series data of physiological indicators, and textual information of patient history. First, the data collection and preprocessing phases aim to obtain multimodal medical information, including CT images, physiological indicator time series and patient history text. These data were professionally cleaned and standardized to ensure data quality and consistency. Simultaneously, privacy protection measures are employed to ensure the security of sensitive patient information, and this step aims to prepare multimodal data for model training and evaluation.

**FIGURE 1:**
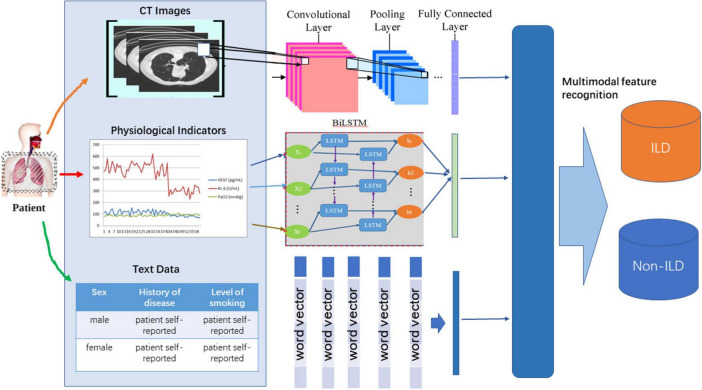
Diagram of the overall implementation framework of the proposed ILDIM-MFAM model.

As shown in Figure 1, MFAM was introduced to fuse different modalities of medical information with the aim of fusing features from CT images, physiological indicators, and textual information to capture the multifaceted features of ILD. MFAM achieves the optimal fusion by learning the importance weights of each modality of data to improve the diagnostic performance of ILD. Convolutional Neural Network (CNN) is used to extract features from CT image slices. The purpose of this step is to capture the morphological and structural features of ILD images to provide rich visual information for the model. Bidirectional Long Short-Term Memory Network (Bi-LSTM) is used to learn long-term dependencies in time-series data of physiological indicators. The purpose of this step is to extract time-dependent features from time-series data to better understand ILD trends. Self-attention mechanism is used to encode textual information in patients’ self-reports and medical prescriptions. The purpose of this step is to transform the textual information into meaningful semantic representations for use in the model. The multimodal perception mechanism uses a Transformer-based model that fuses data from different modalities and learns the importance weights of each modality to optimally fuse this information. This helps to improve the diagnostic performance of ILD.

**B. Subject selection and clinical data collection**

We selected patients attending the Respiratory Department of the First Hospital of Gannan University of Medical Sciences as the study population. Patients with IPF-ILD were randomly selected into the study group, while patients with other non-ILD diseases were included in the control group. All studies were conducted in accordance with the regulations of the Medical Ethics Committee of our hospital and ethical review was performed. All the study subjects involved in blood collection participated in the study with prior informed consent. The study was approved by the Ethics Committee of the First Affiliated Hospital of Gannan Medical University under the ethical approval number [**LLSC2023-125**].

**C. Data processing and analysis**

It is evident that the majority of individuals suffering from IPF-ILD exhibit notably lower PaO_2_ pulmonary gas function values when compared to non-ILD subjects under identical clinical conditions. This finding underscores the substantial impact of IPF-ILD on pulmonary gas function. While it is worth noting that certain normal individuals can have lower PaO_2_ values than some IPF-ILD patients due to factors such as inherent inter-individual variations, physiological characteristics, and potential testing errors, an overarching trend toward decreased lung gas function is unmistakably evident among IPF-ILD patients. This trend is likely associated with the unique characteristics of the disease and structural alterations within the lungs. Consequently, this discovery underscores the deleterious consequences of IPF-ILD on pulmonary air function and establishes a critical foundation for further research and treatment development (28).

**FIGURE 2:**
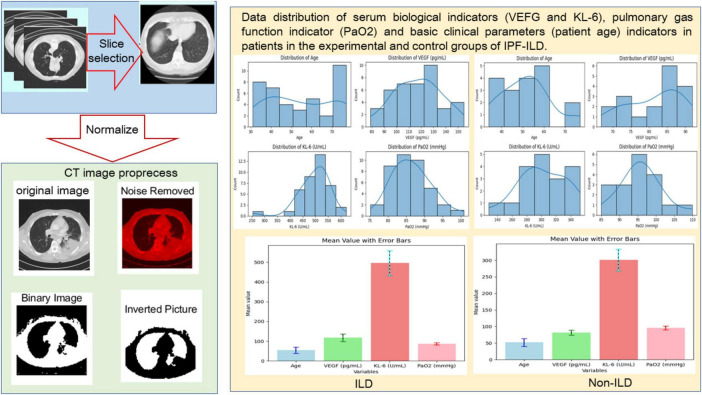
Preprocessing of CT slice data (including manual screening of CT images and image processing techniques and distribution visualization of basic clinical data.

In this study, we selected 40 ILD samples as the experimental group and 20 Non-ILD samples as the control group. The selection of samples strictly followed the pre-set inclusion and exclusion criteria to ensure the reliability and validity of the study results. Specifically, the inclusion criteria included patients with a confirmed diagnosis of idiopathic pulmonary fibrosis (IPF-ILD), who were all treated in the same hospital and had a clear clinical history and imaging findings. Meanwhile, all participants were required to be at least 18 years old and able to cooperate with relevant experimental and clinical assessments. Exclusion criteria included patients with other known lung diseases, individuals who had recently undergone lung surgery, and patients with severe comorbidities such as heart disease or other major medical conditions. In addition, any individuals who had received medications that affected lung function or VEGF and KL-6 levels during the study period were also excluded to minimize potential confounders. Moreover, we collected samples covering patients of different ages, and as can be seen in Figure 2, all sample characteristics conformed to a reasonably good normal distribution, which ensured the applicability of the study results across different demographic characteristics.

In terms of pulmonary function indexes, the PaO_2_ values of IPF-ILD patients were mainly distributed around 85 *mmHg*, while those of the control group were mainly distributed around 95 *mmHg*. This indicates that under the same clinical conditions, IPF-ILD patients have significantly impaired pulmonary gas function, which is reflected in lower PaO_2_ values. In summary, through in-depth analysis of the data in Figure 2, we clarified the differences between IPF-ILD patients and the control group in terms of age, serum VEGF and KL-6 levels, and pulmonary air function indicators.

**D. CT slice image feature extraction via CNN**

CNN is a deep learning model specially designed for image recognition and feature extraction, which is composed by convolutional, pooling and fully connected layers to automatically learn and extract features from images, and has achieved good results in practical applied research (29). In this section, we mainly want to extract features from chest CT image slices by CNN to capture morphological information and structural features of ILD. Convolutional operation is the core of CNN. It extracts local features by sliding a convolution kernel over the image. It can be described as the following Equation 1:


(1) $$\begin{array}{l} Conv\left(I,K\right)\;=\;\left(I*K\right)\;\left(x,y\right)\\ =\Sigma_{i=1}^{M}\Sigma_{j=1}^{N}\;I\left(x-i,y-i\right)\;K\;\left(i,j\right) \end{array}$$

where *I* denotes the input image, *K* is the convolution kernel, *M* and *N* denote the height and width of the convolution kernel, respectively. The convolution operation multiplies and sums the convolution kernel with the input image point by point to generate the convolved output feature map. Moreover, pooling manipulation is used to reduce the spatial size of the feature map, and to reduce the amount of computation, and to extract the most important features.

As shown in Algorithm 1, the pseudo-code describes the process of extracting features from chest CT images via CNN. First, the algorithm accepts the input CT image and convolution kernel and initializes the output feature map. For each image, the algorithm progressively performs a convolution operation to compute the output value at each position by sliding the convolution kernel over the image. Moreover, a pooling operation is applied to reduce the spatial size of the feature map and extract the maximum value of each region, and finally the processed feature maps are summarized as output. This process aims to capture the morphological features and structural information of the image to provide important visual input for subsequent disease diagnosis.

**ALGORITHM 1:** Feature extraction from chest CT images using CNN.

**Input:** Input images **I** (chest CT image slices), convolution kernel K **Output:** Reduced feature maps **F** after convolution and pooling **1:** Initialize output feature maps **F** ←□ **2:** **for** each image I in **I** **do** **3:** // Step 1: Perform Convolution **4:** Initialize outputMap as a matrix of appropriate size **5:** **for** each position (x,y) in I **do** **6:** **outputMap**[x, y] ←$\sum_{i=0}^{M-1}\sum_{j=0}^{N-1}I\,\left[x+i,y+j\right]\,K\,\left[i,j\right]$ **7:** **end for** **8:** // Step 2: Apply Pooling **9:** Initialize **pooledMap** of reduced size **10:** **for** each region in outputMap **do** **11:** **pooled Value** ← max **(value** in region) **12:** Assign **pooledValue** to the corresponding position in **pooledMap** **13:** **end for** **14:** Append **pooledMap** to **F** **15:** **end for** **return F**	

**E. Time-series feature extraction of physiological indicators via Bi-LSTM**

The purpose of this section is to extract features from time-series data of physiological metrics by means of a Bi-LSTM to capture the temporal dependencies and trends of changes in ILDs as manifested in the patient’s physiology. It is a deep learning model for sequence data to capture long-short term dependencies in sequences. The Bi-LSTM consists of two LSTM layers, one propagating sequence from the front and the other propagating sequences from the back, and finally their outputs are merged together. In which LSTM is a variant of Recurrent Neural Network (RNN) for processing sequence data (30, 31). LSTM consists of an input gate, forgetting gate, output gate and cell state that learns and stores the information in the sequence.

As shown in Algorithm 2, the pseudo-code describes the process of extracting features from time-series data of physiological metrics using a bi-directional long- and short-term memory network (Bi-LSTM). First, the hidden and cellular states of the forward and backward LSTMs are initialized. For each time step, input gates, forget gates, output gates, and candidate cell states are computed, and the cell states are updated based on the outputs of these gates. Then, the final feature representation is generated by merging the hidden states of the forward and backward LSTMs. This process can effectively capture the long- and short-term dependencies in time-series data and provide important temporal feature information for the analysis of ILD.

**ALGORITHM 2:** Feature extraction from time-series data using Bi-LSTM.

**Input:** Time-series data X = [x_1_, x_2_,…, x_*T*_], weight matrices W_*i*_, W_*f*_, W_0_, biases b_*i*_, b_*f*_, b_0_ **Output:** Hidden states H = [h_1_, h_2_,…, h_*T*_], cell states C = [c_1_, c_2_,…, c_*T*_] **1:** Initialize C_0_ <— 0, H_0_ <— 0 **2:** **for** time step t = 1 to **T do** **3:** **Forward LSTM:** **4:** Compute input gate: i_*t*_ = σ(W_*i*_ [h_*t*–1_, x_*t*_] + b_*i*_) **5:** Compute forgetting gate: f_*t*_ = σ(W_*f*_ [h_*t*–1_, x_*t*_] + b_*f*_) **6:** Compute output gate: o_*t*_ = σ (W_*o*_ [h_*t*–1_, x_*t*_] + b_*o*_) **7:** Compute candidate cell state: C_*t*_ = tanh(W_*c*_ [rit_i,x_*t*_] + b_*c*_) **8:** Update cell state: C_*t*_ = f_*t*_ C_*t*–1_ + i_*t*_ C_*t*_ **9:** Compute hidden state: h_*t*_ = o_*t*_ tanh(C_*t*_) **10:** **Backward LSTM:** **11:** Compute input gate (backward): i′_*t*_ = σ(W*_*i*_* [h_*t+1*_, x_*t*_] + b_*i*_) **12:** Compute forgetting gate (backward): f′_*t*_ = σ(W_*f*_ [h_*t+1*_, x_*t*_] + b_*f*_) **13:** Compute output gate (backward): o′_*t*_ = σ(W_*o*_ [h_*t+1*_, x_*t*_] + b_*o*_) **14:** Compute candidate cell state (backward): C′_*t*_ = tanh(W_*c*_ [h_*t+1*_, x_*t*_] + b_*c*_) **15:** Update cell state (backward): C′_*t*_ = f′_*t*_ C′_*t*+1_ + i′_*t*_ C′_*t*_ **16:** Compute hidden state (backward): h′_*t*_ = o′_*t*_ tanh(C′_*t*_) **17:** Merge outputs: H_*t*_ = h_*t*_ + h′_*t*_ **18:** **end for** **return** H, C	

In particular, the input gate controls the extent to which new information flows into the cell state. It determines which information should be added to the cell state by means of a *Sigmoid* activation function. The formula for the input gate is described as Equation 2:


(2) $$i_{t}=\sigma \,\left(W_{i}●\left[h_{t-1},x_{t}\right]+b_{i}\right)$$

where *i*_*t*_ is the output of the input gate, σ denotes the Sigmoid activation function, *W*_*i*_ is the weight matrix, *b*_*i*_ represents the bias of the input gate, *h*_*t*–1_ is the hidden state of the previous time step, and *x*_*t*_ is the input of the current time step.

The output of the forgetting gate determines which parts of the cell state from the previous time step should be retained or discarded. The formula for the forgetting gate is described as Equation 3:


(3) $$f_{t}=\sigma \,\left(W_{f}●\left[h_{t-1},x_{t}\right]+b_{f}\right)$$

where *f*_*t*_ represents the output of the forgetting gate, *W*_*f*_ is the weight matrix, and *b*_*f*_ is the bias of the forgetting gate. LSTM passes information through the cell state. The formula for updating the cell state is described as Equation 4:


(4) $$c_{t}=f_{t}●c_{t-1}+i_{t}●{\hat{c}}_{t}$$

where *c*_*t*_ represents the cell state at the current time step, *f*_*t*_ is the output of the forget gate, *c*_*t*–1_ is the cell state at the previous time step, *i*_*t*_ is the output of the input gate, and ${\hat{c}}_{t}$ is the new candidate cell state. The LSTM output gate controls which information from the cell state can be output to the hidden state. The specific formula is described as Equation 5:


(5) $$o_{t}=\sigma \,\left(W_{o}●\left[h_{t-1},x_{t}\right]\right)+b_{o}$$

where *o*_*t*_ represents the output of the output gate, *W*_*o*_ is the weight matrix, *h*_*t*–1_ is the hidden state of the previous time step, and *x*_*t*_ is the input of the current time step, *b*_*o*_ is the bias of the output gate.

The hidden state of the LSTM is the output of the current time step, which is jointly determined by the output gate and the cell state. The specific formula is described as Equation 6:


(6) $$h_{t}=o_{t}●\tanh\left(c_{t}\right)$$

where *h*_*t*_ represents the hidden state of the current time step, *o*_*t*_ is the output of the output gate, *c*_*t*_ is the cell state of the current time step, and **tanh** denotes the hyperbolic tangent activation function.

***F. Text Semantic Extraction via self-attention mechanism***

The purpose of this section is to extract semantic features from textual data by means of Self-Attention Mechanism (SAM) in order to capture important information related to ILD in the textual information of patient medical history. The Self-Attention mechanism is a powerful technique for text processing and sequence modeling that automatically learns to assign different weights to information at different positions in a sequence based on the context of the input sequence (32).

The SAM allows the model to dynamically assign weights to each word while processing a text sequence in order to focus on important words. Attention Scores in SAM can be calculated for each word by Equation 7:


(7) $$E_{i,j}=\frac{\left(Q_{i}●{\left(K_{j}\right)}^{T}\right)}{\sqrt{d_{k}}}$$

where *E*_*i,j*_ denotes the attention score, *Q*_*i*_ denotes the query vector, *K*_*j*_ denotes the key vector, and *d*_*k*_ denotes the dimension of the key vector. After that, Attention Weights are calculated by Equation 8:


(8) $$A_{i,j}=S\,o\,f\,t\,m\,a\,x\,\left(E_{i,j}\right)$$

where *A*_*ij*_ denotes the attention weights and **Softmax** function is used to normalize the attention scores to a probability distribution. Thus the Self-Attention Output (SAO) can be calculated by Equation 9:


(9) $$S\,A_{i}=\sum_{j}\left(A_{i,j}●V_{j}\right)$$

where *SA*_*i*_ denotes the self-attentive output and *V*_*j*_ denotes the value vector. The employed multi-head self-attention allows the model to learn multiple sets of different self-attention weights to capture different types of relations and semantics, which can be described as Equation 10:


(10) $$M\,H\,S\,A_{i}=\left[S\,A_{1\,i},\,S\,A_{2\,i},\cdots ,\,S\,A_{H^{i}}\right]●W_{o}$$

where *MHSA*_*i*_ denotes the multi-head self-attentive output, *H* denotes the number of attention heads, and *W*_*O*_ denotes the output weight matrix. With the above formula, the self-attention mechanism is able to dynamically learn the importance of different words according to the context of the text sequence and extract semantic features, which helps to capture the information related to ILD.

As shown in Algorithm 3, the pseudo-code describes the process of extracting semantic features from text data by SAM. First, the input is a sequence of text and the corresponding query, key and value vectors. Then, by calculating the attention scores, the model can evaluate the importance of different words in the text. The attention weights for each word are normalized by a **Softmax** function to form a probability distribution. Then, using these weights, the model computes the self-attention output to extract the key information related to the ILD. In addition, the use of a multi-head self-attention mechanism enables the model to learn different semantic relationships and features, enhancing the understanding of textual information. This process helps the model to capture semantic information related to ILD more efficiently to support subsequent diagnosis.

**ALGORITHM 3:** Semantic feature extraction using self-attention mechanism.

**Input:** Input text sequence X = [x_1_,x_2,_…, x_*n*_], query vectors **Q,** key vectors K, value vectors **V,** number of attention heads H **Output:** Self-attention output SAO **1** Initialize attention scores E ← 0 for all i, j **2** **for** each word j in X do **3** ** for** each word **i** in X do **4** Compute attention score: E_*i,j*_←$\frac{Q_{i}\,K_{j}^{T}}{\sqrt{d_{k}}}$(Eq. 7) **5** end **for** **6** end **for** **7** Initialize attention weights A ← 0 for all i, j **8** **for** each word i in **X do** **9** Compute attention weights: A_*i,j*_ ← Softmax(E_*i,j*_) (Eq. 8) **10** end **for** **11** Initialize self-attention output SAO ← 0 **12** **for** each word i in **X do** **13** ** for** each word j in X do **14** Compute self-attention output: SA_*i*_ ←$\sum_{j=1}^{n}A_{i,j}\,V_{j}$ (Eq. 9) **15** end **for** **16** end **for** **17** Initialize multi-head self-attention output MHSA ← 0 **18** **for** each attention head h = 1 to H do **19** Compute multi-head self-attention output: MHSA_*i*_ **←** SA_*i*_ W_0_ (Eq. 10) **20** end **for** **return** MHSA

**G. Transformer model with multi-modal perception**

**FIGURE 3:**
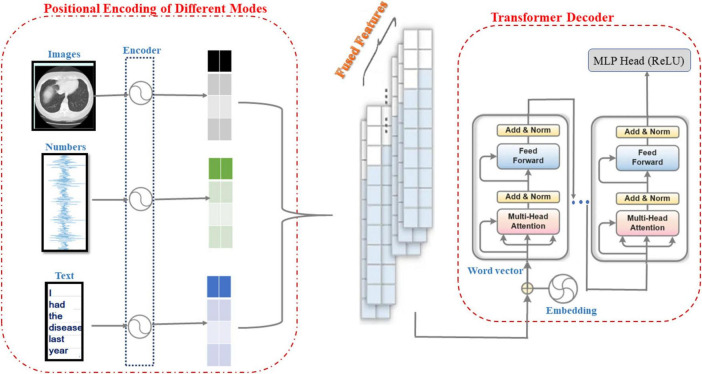
Schematic of the fusion of different model features.

As shown in Figure 3, the information from CT image modality, physiological metrics data modality and textual semantic modality are positionally encoded by encoders, and the Transformer model is used to sense the signals from these three modalities and perform feature fusion and pattern perception. Therefore, the main purpose of the research in this subsection is to develop a robust multimodal perception system based on the Transformer model that is capable of extracting high-level semantic features from medical information data in different modalities to support accurate diagnosis and classification of ILD. First, the information from different modalities (e.g., images, temporal data, and text) is effectively combined to obtain a more comprehensive representation of ILD features. Moreover, through Transformer’s self-attention mechanism, the model can automatically learn and understand the correlations and dependencies between different modalities to better recognize the relevant information of ILD. Finally, the Transformer model’s multi-head self-attention mechanism can capture long-range dependencies in multimodal data and extract high-dimensional semantic features, which helps to recognize ILD more accurately.

As shown in Algorithm 4, this pseudo-code describes the construction process of a multimodal sensory system based on the Transformer model. First, the system receives CT images, time-series physiological metrics, and textual semantic data as inputs and performs feature extraction and encoding for each modality by means of the CNN, Bi-LSTM, and the Transformer encoder. Next, the fusion weights of each modality are calculated to dynamically determine their contributions in the fusion. The associations between different modalities are learned via MHSA to generate a multimodal fusion feature representation. The fused features are then fed into the Transformer encoder to generate the model output. During model training, the loss is calculated and the model parameters are updated using the **AdamW** optimizer, which ultimately allows prediction based on new CT images and clinical data.

**ALGORITHM 4:** Multimodal perception system using transformer.

**Input:**		
	CT image data **X**_*image*_, physiological metrics time-series data X_*num*_, textual semantic data **X**_*text*_	
**Output:**		
	Multimodal fused feature representation **M**, model output **Y**	
1	**Encode each modality:**	
2	H_*i*_ ← CNN(**X**_*image*_)	⊳ Extract features from CT images
3	H_*n*_ ← Bi-LSTM(**X**_*num*_)	⊳ Model time-series data
4	H_*t*_ ← Transformer_Encoder(**X**_*text*_)	⊳ Encode textual data
5	**Compute fusion weights:**	
6	W_*i*_, W_*n*,_ W_*t*_ ← calculate_weights(H_*i*_, H_*n*_, H_*t*_)	
7	**Modality-specific feature representations:**	
8	**a_*i*_** ← **W_*i*_ H_*i*_**	⊳ CT image feature
9	**a_*n*_** ← **W_*n*_ H_*n*_**	⊳ Numerical data feature
10	**a_*t*_** ← **W_*t*_ H_*t*_**	⊳ Text data feature
11	**Multimodal Feature Fusion (MMFF):**	
12	MF←MHSA(a_*i*_, a_*n*_, a_*t*_)	⊳ Learn correlations between modalities
13	**Compute fused feature representation:**	
14	**M** ← weight_fused_features (MF, **W_*i*_, W_*n*_, W_*t*_)**	
15	**Model output:**	
16	**Y** ← Transformer_Encoder(M)	
17	**Loss computation:**	
18	**L**<— cross_entropy_loss(Y, Y_*k*_)	
19	**Optimize model parameters:**	
20	Update parameters θ← AdamW(L, θ)	⊳ Using AdamW optimizer
21	**Make predictions for new data:**	
22	Given new data (X_1_, X_2_, X_3_), compute Y ← *M*(X_1_, X_2_, X_3_)	
	**return Y**	

For images, the CNN is used to extract features. For numerical data, the Bi-LSTM is used for sequence modeling. For text data, Transformer encoder is adopted for encoding. The input data for each modality is encoded to obtain modality specific feature representation. Assume that the output of the image encoder is *H*_*i*_, the output of the numeric data encoder is *H*_*n*_, and the output of the text data encoder is *H*_*t*_, the fusion weights between each modality are computed in order to dynamically determine the contribution of each modality to the fusion. The feature representations for each modality are described as Equations 11–13:


(11) $$\alpha_{i}=E\,\left(W_{i}*H_{i}\right)$$

(12) $$\alpha_{n}=E\,\left(W_{n}*H_{n}\right)$$

(13) $$\alpha_{t}=E\,\left(W_{t}*H_{t}\right)$$

where, *a*_*i*_, *a*_*n*_, *a*_*t*_ are the coded features of CT images, physiological indicator time-series data and textual semantics, respectively, and *W*_*i*_, *W*_*n*_, *W*_*t*_ are the coded weights, respectively. Moreover, Multimodal Feature Fusion (MMFF) uses Multi-Headed Self-Attention (MHSA) to learn the correlation between each modality and generate the weight matrix by Equation 14:


(14) $$M\,F\,\left(\alpha_{i},\alpha_{n},\alpha_{t}\right)=S\,A\,\left(\left[\alpha_{i},\alpha_{n},\alpha_{t}\right]\right)$$

where *MF* denotes multimodal fusion and *SA* denotes self-attention mechanism. Furthermore, the computation of individual modal fusion weights is realized by the self-attention mechanism, which is used to learn the weights of each modality by Equation 15.


(15) $$w_{i}=\frac{\exp\left(E_{i}\right)}{\sum_{j}\exp\left(E_{j}\right)}$$

where *w*_*i*_ denotes the attention weight and *E*_*i*_ denotes the attention score calculated from the self-attention mechanism.

The multimodal fused feature representation is input to the Transformer encoder for further capturing the relationship between different modalities. The weights calculated earlier can be used to weight the fused feature representations of different modalities by Equation 16:


(16) $$M=\sum_{i}w_{i}●\beta_{i}$$

where *M* denotes multimodal fusion, and β_*i*_ denotes the feature representation after encoding different modalities. The output $\hat{Y}$ of the model can be described as Equation 17:


(17) $$\hat{Y}=S\,o\,f\,t\,m\,a\,x\,\left(\lambda ●M+b\right)$$

where $\hat{Y}$λ, *b* are the learning parameters. Furthermore, the cross-entropy loss to measure the difference between the model’s predictions and the true labels can be described as Equation 18.


(18) $$\ell =-\sum_{k=1}^{K}Y_{k}●\log\left({\hat{Y}}_{k}\right)$$

where *Y*_*k*_ is the one-hot encoding of the real label. After that, the gradient descent or its variants are used to minimize the loss function from Equation 19:


(19) $$\theta^{*}=\underset{\theta }{\arg\min}\ell \,\left(\theta \right)$$

where θ denotes the parameters of the model. The gradient of the loss function with respect to the parameters is computed by the back-propagation algorithm. Moreover, the **Adamw** optimizer is used to update the model parameters. After training is complete, we can use the trained multimodal model to make predictions. Given new CT images *X*_1_ and clinical data *X*_2_, as well as textual semantic information about doctors and patients *X*_3_, we can compute the output *Y* of the model by Equation 20:


(20) $$Y=\underset{k}{\arg\max}{\hat{Y}}_{k}$$

## 3 Results

Ablation experiments are necessary to validate the performance benefit under different modal inputs and to explore the contribution of data characteristics of different modalities to the diagnostic performance of ILD. In this study, CT images with detailed information characterizing the structure and morphology of the lungs, data on physiological indices reflecting lung function and gas exchange, and textual information providing a description of the patient’s background and condition were used as inputs for multi-modal signals to explore the correlation between different modal information and ILD.

Therefore, the design of this multimodal ablation test is based on the fact that ILD is a complex lung disease and its diagnosis requires multifaceted information. Using any one data source alone can lead to insufficient information or misdiagnosis. Combining text, CT images, and physiologic indicators can provide more comprehensive, multifaceted information that can help accurately diagnose the type and severity of ILD and the overall health of the patient.

**A. Analysis of experimental results**

**FIGURE 4:**
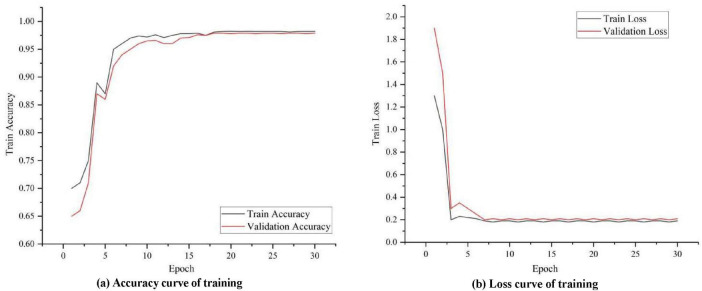
Visualization of the training and learning process of the proposed ILDIM-MFAM model.

To observe how well the proposed model learns on image data, temporal data and textual semantic data, the accuracy and loss during its training and validation are visualized in Figure 4. It can be seen that in the initial stage, the training accuracy increases rapidly and the model learns some basic patterns and features. As training progresses, the accuracy curve can gradually level off, indicating that the model has largely converged to the optimal solution. Eventually, the training accuracy curve can stabilize at a relatively high level, indicating that the model is performing well on the training data. The validation accuracy curve is similar to the training accuracy curve in the initial phase, but gradually stabilizes in subsequent phases. After the validation accuracy stabilizes, it should be close to or consistent with the training accuracy curve, indicating that the model also performs well on unknown data without overfitting.

**FIGURE 5:**
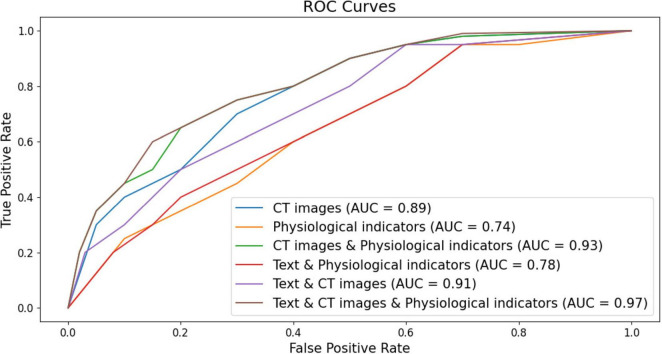
Classification ROC curves with six different forms of modal inputs (respectively, CT images, CT images & physiological indicators, CT images & physiologic indicators, Text & physiological indicators, Text & CT images, Text & CT images & physiologic indicators for the six different forms of modality).

As shown in Figure 5, the ROC curves of the ILD diagnostic performance under six different modal forms of input are elaborated, and overall, the information from each modality is seen to be an effective way to diagnose ILD. It can be seen that the model using only CT image data for ILD diagnosis has an accumulated area under the ROC curve (AUC) of 0.89. This implies that the model has a good ability to differentiate between ILD and non-ILD situations and its performance is relatively high. The model for ILD diagnosis when using only physiologic indices accumulated an area under the ROC curve (AUC) of 0.74. Although the AUC value is smaller than the other models, it still indicates that physiologic indices have the ability to discriminate between the ILD. It is worth mentioning that the model combining CT images and physiologic indicators achieved an AUC value of 0.93, showing a high ILD diagnostic performance. This suggests that combining information from multiple modalities can significantly improve the accuracy of ILD diagnosis and that there can be complementarity between the two data sources. Moreover, the model combining text data and CT images achieved an AUC value of 0.91, indicating that these two data sources have significant value in ILD diagnosis. The combination between text data and CT images improved the performance of the model. However, combining text data, CT images, and physiologic index information for ILD diagnosis showed excellent performance with an AUC of 0.97. This result suggests that combining multimodal data sources can achieve very high ILD diagnostic accuracy, which is important for clinical decision making.

**FIGURE 6:**
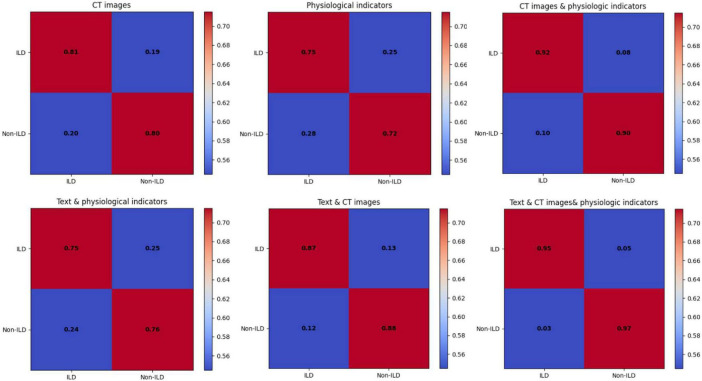
Classification performance analysis of ILD diagnostic models with different modal inputs.

The data of the detailed ablation test results for each index are statistically presented in Table 1, which shows that the model performance using CT images performs relatively well with high Precision and Recall in the journals with unimodal inputs. This suggests that CT images can provide important information about ILD characteristics and help to differentiate between ILD and Non-ILD cases. The CT images can capture details of lung structures and lesions, and therefore have an important role in diagnosis. When combining the input modalities of CT images and physiological indicators, the performance of the model was improved in all aspects, including precision, recall, F1 score, and AUC. This suggests that by fusing multimodal information, the model can better synthesize the information from CT images and physiological indicators, which improves the comprehensive performance, and that this joint modeling enables a more comprehensive assessment of the patient’s health status. Although the performance metrics in the case of using the combined modality of text and physiological indicators are slightly lower than the case of using CT images alone, they are still relatively good. The better performance of the model combining textual and physiological indicator modalities than using physiological indicator modalities alone suggests that the semantic information of textual modalities still has a non-negligible role in diagnosis, especially when the model needs to take into account aspects such as the patient’s clinical history, symptom description, and so on. The model achieved the highest level of performance with the highest precision, recall, F1 score, and AUC when simultaneously fusing CT images, textual information, and physiological metrics. As shown in Figure 6, which exhibits the confusion matrix of the model test results for different modal inputs, the visualization results once again emphasize the importance of multimodal information fusion. It is very obvious to see that combining information from different modalities allows for a more comprehensive assessment of the patient’s condition and improves the diagnostic comprehensiveness of ILD.

**TABLE 1:** Statistics of diagnostic metrics with different forms of modal inputs

Input modes	Precision	Recall	F1	AUC
CT images	0.85	0.80	0.82	0.89
Physiological indicators	0.72	0.65	0.68	0.74
CT images & physiologic indicators	0.90	0.88	0.89	0.93
Text & physiological indicators	0.75	0.70	0.72	0.78
Text & CT images	0.88	0.84	0.86	0.91
**Text & CT images& physiologic indicators**	**0.94**	**0.95**	**0.94**	**0.97**

In summary, the results of the ablation trial emphasize the importance and complementarity of the different input modalities in the diagnosis of ILD. Among them, CT images provide important structural information, physiologic indicators provide physiologic information, and textual information provides clinical context and symptom description. The combined utilization of this information can help improve the comprehensive performance of ILD and provide more accurate diagnosis and treatment for patients. The fusion of different modality information can better meet the complexity and diversity needs of medical diagnosis.

## 4 Discussion of the application potential of the ILDIM-MFAM

The previous ablation experiments comparatively analyzed the excellent performance of the ILD diagnostic study proposed in this study in terms of validity and engaging accuracy of different modal fusions. To analyze the comprehensive diagnostic performance of the ILDIM-MFAM model proposed in this study, as well as the potential for practical improvements and applications, the diagnostic accuracy and the computational complexity of the model were considered comprehensively.

As shown in Table 2, The ILDIM-MFAM model is designed to effectively address the identification of interstitial lung diseases by integrating multi-modal data sources through a sophisticated attention mechanism. By leveraging a combination of convolutional and recurrent neural networks alongside transformer-based attention mechanisms, the model adeptly captures intricate features across diverse data modalities, thereby enhancing both performance and efficiency. The fusion of information from various modalities enables comprehensive representation learning, while the attention mechanism selectively focuses on relevant features, optimizing both predictive accuracy and computational efficiency. This design approach ensures that the ILDIM-MFAM model achieves a balance between performance and efficiency, making it well-suited for practical deployment in clinical settings where accurate and timely identification of interstitial lung diseases is paramount.

**TABLE 2:** Parameter statistics of the proposed ILDIM-MFAM model.

Layer (type)	Output shape	Param #
Input	(32, 16, 1024)	0
Conv2d (in_channels = 3, out_channels = 64)	(64, 112, 112)	1792, 000
ReLU	(64, 112, 112)	333,280
MaxPool2d (kernel_size = 2)	(64, 56, 56)	0
Bi-LSTM (input_size = 10, hidden_size = 20)	(1024, 20)	183,024
TransformerEncoderLayer	(1024, 64)	1280, 000
Linear (Attention Weights)	(32, 16, 256)	69,120
Linear (Attention Weights)	(1, 10)	2,570
Softmax	(1, 10)	0
Linear (Classifier)	(2, 10)	2100
Total params: 4.67 M		
Trainable params: 4.54 M		
Non-trainable params: 0 M		
Input size: 2.10 M		
Forward/backward pass size: 0.12 M		
Params size (MB): 4.7 M		
Estimated Total Size: 4.71 M		

# Indicates a comment symbol for the given pseudo-code.

**FIGURE 7:**
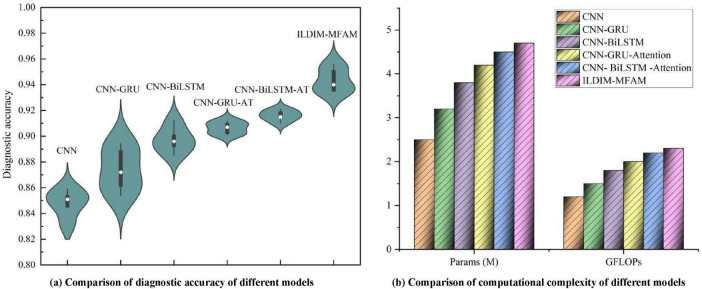
The ILD diagnostic accuracies of different models after inputting multimodal data, and the comparison of their model parameters and computations [in panel **(a)**, the diagnostic accuracies of 5 tests were used for statistical analysis in order to make the test results of the models more robust. In panel **(b)**, the number of parameters and complexity of different models are used to synthesize the application value of ILD diagnostic models].

As shown in Figure 7, multiple deep learning decoding models, which were used to diagnose ILD & Non-ILD, with the aim of evaluating their performance in the presence of multimodal data inputs as well as the trade-off with computational complexity. By comparing the performance of the different models, it can be seen in Table 3 that the proposed ILDIM-MFAM shows the best results in various performance metrics (Precision, Recall, F1) and achieves an F1 score of 0.94, which indicates that the model has a high level of accuracy and comprehensiveness in the task of classification of ILD and Non-ILD. This is in line with the main goal of the study, which is to improve the accuracy of ILD diagnosis. In addition, as can be seen in part (a) of the figure, ILDIM-MFAM also performs well in terms of robustness by statistically analyzing the five tests. This means that the model’s performance is relatively stable across different datasets or test sets and is not susceptible to randomness, which is important for clinical applications.

**TABLE 3:** Existing state-of-the-art deep learning decoder models with multimodal features input are used for performance comparison.

Model	Precision	Recall	F1	Params (M)	GFLOPs
CNN	0.85	0.88	0.86	2.5	1.2
CNN-GRU	0.88	0.89	0.88	3.2	1.5
CNN-Bi-LSTM	0.90	0.92	0.90	3.8	1.8
CNN-GRU-Attention	0.91	0.93	0.91	4.2	2.0
CNN- Bi-LSTM -Attention	0.92	0.94	0.91	4.5	2.2
**The proposed ILDIM-MFAM**	**0.94**	**0.95**	**0.94**	**4.7**	**2.3**

In terms of computational complexity, the results in Table 3 demonstrate that ILDIM-MFAM has a relatively low number of model parameters with 4.7 M and computational complexity of Giga Floating-point Operations Per Second (GFLOPs) with 2.3, respectively. Although it performs well in terms of performance, the relatively low number of model parameters and computational complexity make ILDIM-MFAM more efficient in terms of computational resources. This is very favorable for real-world deployment and runtime efficiency. The superior performance of these studies is mainly due to the fact that ILDIM-MFAM employs the Transformer principle, which is very beneficial for multimodal data processing. The Transformer model can handle correlation and information fusion between different modalities, which is especially important in the case of multimodal data input. Through the self-attention mechanism and multi-head mechanism, Transformer can adaptively capture key information in the input data, which improves the model’s ability to adapt to different data modalities.

Although the proposed ILDIM-MFAM model exhibits excellent performance and computational efficiency, there are still some potential drawbacks and room for improvement. First, the model performance can be affected by the amount of data, especially in the context of scarce medical data, and the model can lose some of its accuracy. Second, considering the clinical reality, medical data can contain noise and uncertainty, so the robustness and generalization ability of the model still need to be further enhanced. In addition, the interpretability of the models is also a challenge, as the internal working mechanisms of deep learning models are often difficult to explain to healthcare practitioners and patients. Future work could focus on expanding and diversifying the collection of datasets to enhance the robustness of the models, as well as investigating more interpretable methods of model interpretation to improve the acceptance of medical applications. In addition, more migration learning and continuous monitoring methods can be explored to ensure that the model can adapt to changes in different medical scenarios and time dimensions to improve its usefulness.

## 5 Conclusions and discussion

It is worth noting that although nintedanib is currently approved for the treatment of progressive pulmonary fibrosis (PPF), we need to emphasize the importance of accurate diagnosis (33, 34). In particular, in some patients with fibrotic interstitial lung diseases (ILDs) with non-idiopathic pulmonary fibrosis (non-IPF), a combination of immunosuppressive and antifibrotic agents may be required to optimize treatment outcomes. Available clinical studies have shown that some patients respond favorably to combination therapy in specific situations. Therefore, accurate identification of the type of pathology in these patients is essential for the development of a personalized treatment plan. This will not only improve patient prognosis but also guide future clinical decisions.

The purpose of this study is to address the challenges in the field of interstitial lung disease (ILD) diagnosis and develop an innovative ILD identification model (ILDIM) by introducing a multimodal fusion attention mechanism (MFAM). We have fully utilized multimodal medical data such as chest CT image slices, physiological index time series data, and textual information of patient history to characterize the patient’s health status in a more comprehensive and multifaceted way. The main contributions of this study include solving the multimodal medical data fusion problem, using an attention mechanism to adaptively fuse different modal information, and comprehensively considering the multidimensional features of ILD diagnosis, thus significantly improving the diagnostic comprehensiveness and accuracy of ILD. It is proved through experiments that the model performs well in terms of comprehensive performance, which not only improves the accuracy, recall and F1 score, but also significantly increases the AUC value. This implies that the combined use of different modal information can assess the health status of patients more comprehensively and provide more reliable support for ILD diagnosis.

Although MFAM performs well in multimodal data fusion, there is room for improvement. Future work could explore more complex modality fusion strategies to further improve model performance. In addition, this study focused on ILD diagnosis, but the approach of multimodal medical information fusion can be extended to other medical fields, such as tumor diagnosis and neurological disease research. Finally, we encourage future studies to explore practical clinical applications to introduce the model into medical practice to provide physicians with better decision support tools, thereby improving patients’ health and quality of life. In conclusion, this study provides strong evidence for the application of multimodal medical information fusion in ILD diagnosis and points the way to future research directions.

It cannot be ignored that this study has some limitations in terms of data collection and model generalization. First, the sample selection was mainly from a single hospital, which may have resulted in an under-representative sample and limited the generalizability of the findings. In addition, the sample size, although sufficient for preliminary analysis, may not adequately reflect the diversity of diseases in different populations. Therefore, future studies should be conducted in a wider range of clinical settings to validate the applicability of the model in different patient populations. Meanwhile, bias, such as selectivity bias and reporting bias, may exist during data collection, and these factors may affect the reliability of the results. Therefore, further studies should consider these limitations to enhance the clinical utility and generalizability of the model.

## Data Availability

The original contributions presented in this study are included in this article/Supplementary material, further inquiries can be directed to the corresponding authors.
